# Primary Atomic Frequency Standards at NIST

**DOI:** 10.6028/jres.106.004

**Published:** 2001-02-01

**Authors:** D. B. Sullivan, J. C. Bergquist, J. J. Bollinger, R. E. Drullinger, W. M. Itano, S. R. Jefferts, W. D. Lee, D. Meekhof, T. E. Parker, F. L. Walls, D. J. Wineland

**Affiliations:** National Institute of Standards and Technology, Boulder, CO 80305-3328

**Keywords:** atomic clock, atomic frequency standard, cesium-beam frequency standard, cesium-fountain frequency standard, clock, fountain frequency standard, frequency, frequency standard, ion frequency standard, laser-cooling of atoms, stored-ion frequency standard, time

## Abstract

The development of atomic frequency standards at NIST is discussed and three of the key frequency-standard technologies of the current era are described. For each of these technologies, the most recent NIST implementation of the particular type of standard is described in greater detail. The best relative standard uncertainty achieved to date for a NIST frequency standard is 1.5×10^−15^. The uncertainties of the most recent NIST standards are displayed relative to the uncertainties of atomic frequency standards of several other countries.

## 1. Introduction

The unit of time interval (the second) and the keeping of time depend on primary frequency standards maintained by a number of the world’s national standards laboratories. The concept of time involves an arbitrary starting point (origin), and it is only time interval or frequency that can really be measured. The second plays a pivotal role as a base unit in the International System of Units, universally abbreviated SI (from the French name *Le Système International d’Unités*). The meter is now directly defined in terms of the second [1], and representations of the volt are maintained through the Josephson effect as a constant times frequency [2]. Furthermore, because frequency can be measured easily with very low uncertainty, many other measurements are often transduced to frequency where simple counting then directly converts the results into digital form. Thus, the development and maintenance of frequency standards has been a high-priority activity at NIST (formerly NBS, the National Bureau of Standards) for many years.

Work on frequency standards at NIST began in 1911 with J. H. Dellinger’s development of a system for calibrating wavemeters. He obtained frequency from a simple calculation of the resonance of an LC circuit. During the next few years, the development of better mathematical expressions for inductance and capacitance provided for considerable improvement in frequency measurements using these types of standards [3]. In the mid-1920s the Bureau began studies of quartz-crystal oscillators as frequency standards and by 1935 had established a national primary standard of radio frequency using a set of four quartz oscillators calibrated against the mean solar second [4]. The end of the era of quartz frequency standards began in 1949 with the development at NBS of the world’s first atomic frequency standard based on an ammonia absorption line at 23.87 GHz [5]. At the beginning of this early period of development of atomic frequency standards, both quartz and atomic standards were achieving frequency uncertainties of about 2×10^−8^. (Throughout this paper, relative frequency uncertainty Δ*ν*/*ν*_0_, where Δ*ν* is the frequency uncertainty generally estimated at the level of one standard deviation and *ν*_0_ is the frequency of the standard, is used to describe the performance of the various frequency standards. This is also called the relative standard uncertainty.) The Bureau supported work on both technologies for the next decade, but the rapid advances in the accuracy of atomic frequency standards could not be matched by quartz devices, and the work on quartz frequency standards was stopped in 1959. This paper describes NBS/NIST work on three classes of atomic frequency standards: cesium-beam frequency standards, cesium-fountain frequency standards, and stored-ion frequency standards. No effort has been made to provide a comprehensive institutional history of this work, since the early part of this history is well covered in papers by Beehler [6] and Ramsey [7] and in books by Snyder and Bragaw [8] and Passaglia [9].

Although the ammonia frequency standard was developed first, it never played a significant role in supporting frequency measurements at NBS, so it is not described further. In describing work on the three classes of atomic frequency standards, emphasis is placed on the most recent devices and the results achieved with them. In particular, the section on cesium-beam frequency standards emphasizes the most recent device, NIST-7, and only brief coverage is given to the preceding beam standards (NBS-1 to NBS-6).

Several general aspects of the various types of atomic frequency standards, including atomic-state interrogation time, the form of the microwave cavity, and line-center servomechanisms are briefly discussed next. Primary atomic frequency standards are passive devices; the resonance is located by probing the system with an external oscillator that can be tuned across the resonance. Generally, the narrower the linewidth, the less uncertain is the location of the center of the resonance, but noise can also affect the accurate location of the center of the line. Ignoring noise for the moment, the linewidth Δ*ν*_a_ of the atomic resonance at frequency *ν*_0_ is reciprocally dependent upon the time *t*_d_ the atoms spend in the state interrogation region. The fractional linewidth is simply proportional to

$$\frac{\Delta \nu_{a}}{\nu_{0}}\approx \frac{1}{t_{d}\nu_{0}}.$$ (1)

This expression shows that the standard should be operated at as high a frequency as is practical and that the atoms should spend as much time as possible in the region where they are probed by the interrogating field provided by the external oscillator. These simple considerations have guided the development of atomic frequency standards, as will become evident as the various standards are described.

The basic concepts upon which atomic frequency standards are based were developed in the early 1940s by Rabi [10], but Ramsey contributed a major improvement with his method of successive oscillatory fields” [11]. In the literature, this is more often referred to as the “method of separated oscillatory fields.” In Rabi’s early molecular-beam experiments at rf frequencies, the molecules or atoms passed through a single rf excitation region and the results obtained were critically dependent upon the uniformity of the weak magnetic field in this region (the so-called C field is needed to establish a quantization axis for the atoms). In Ramsey’s method the rf excitation field is divided into two spatially separated zones that are driven in phase, so that the atoms are first subjected to a short oscillatory field where a transition is initiated and then drift through the C field to a second region where another short oscillatory field induces a completion of the transition. The effect of this process, now called Ramsey interrogation, is to sharpen the resonance and substantially relax the requirements on the homogeneity of the C field. All primary atomic frequency standards now use this means of interrogation.

While a variety of methods have been used to servo-control a local oscillator to the atomic resonance, most modern atomic standards now use square-wave modulation and digital processing to achieve the lock. In this method, the local oscillator spends a certain period on one side of the resonance where the signal amplitude is measured, and then is shifted to the other side of the line for a similar measurement. The difference between the amplitudes of these two signals is driven to zero by the servo-control system, thus assuring that the center of the resonance is at the midpoint between these two positions. This of course requires that the shape of the resonance be symmetric, a consideration that must be checked during evaluation of the accuracy of the standard.

## 2. Cesium-Beam Frequency Standards

### 2.1 Standards Based on Magnet State Preparation and Detection

**Fig. 1:**
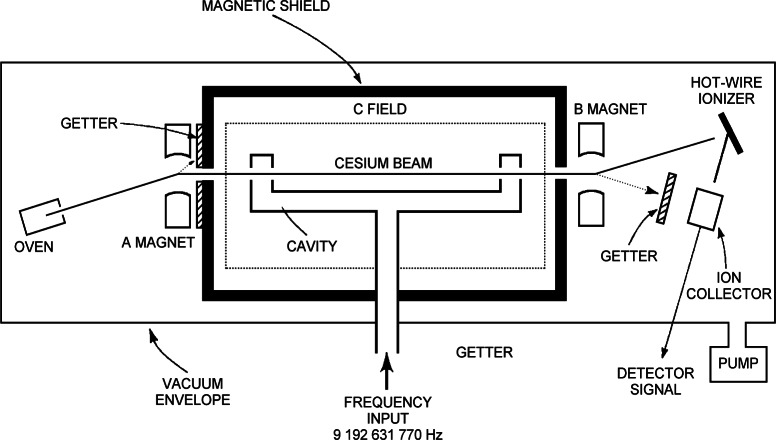
Diagram of a cesium-beam frequency standard using magnetic state selection and detection. The form of Ramsey interrogation involves a U-shaped microwave cavity, called a Ramsey cavity, where the oscillatory fields are spatially separated.

Figure 1 shows a schematic diagram of a conventional cesium-beam frequency standard. The cesium oven, operated near 100 °C, creates a vapor of atoms that are collimated and passed successively through the state-preparation region (the A magnet in Fig. 1), the microwave cavity, and then the state-detection region (the B magnet and detection system). As they emerge from the oven the ^133^Cs atoms are evenly distributed in the 16 *m*_F_ states of the ^2^S_1/2_ ground electronic state. In the state-preparation region, a magnet with an inhomogeneous field (Stern-Gerlach magnet) spatially separates atoms in the various *m*_F_ states, and atoms in one of the ground-state levels (*F* = 3, *m_F_* = 0 or *F* = 4, *m_F_* = 0, often designated as |3,0〉 or |4,0〉) are transmitted through the microwave cavity. Because of the velocity spread in the atomic beam, this separation is not perfect, so some atoms in other *m_F_* states are mixed in with the ground-state atoms that go through the cavity. This type of state preparation naturally involves a rejection of most of the atoms entering the system. State detection uses an identical Stern-Gerlach magnet arranged so that atoms are directed to the hot-wire detector only if they have been stimulated by the microwave field to the other ground, *m_F_* = 0 level.

The designs of the seven NBS/NIST cesium-beam frequency standards, developed between 1950 and 1993, were influenced by a need to reduce and control systematic frequency shifts while maintaining the highest practical signal-to-noise performance. The linewidths of these standards were reduced by extending the length of the microwave cavity, which grew to 3.74 m for NBS-5. This increase in length was achieved at a cost of signal intensity. Furthermore, these long beam tubes, being horizontal, also suffered gravitational dispersion on the order of 1 cm (the slower atoms fall further than the faster ones, which spreads out the beam), thereby complicating the dispersion associated with the magnetic focusing produced by the state-preparation magnet. Reduction of the uncertainty for these beam standards was achieved through a variety of incremental developments, but a gradual improvement in the theory also contributed to improved ways of evaluating and controlling systematic frequency shifts.

### 2.2 NIST-7, An Optically Pumped Cesium-Beam Standard

The use of optical pumping to replace state-selection magnets was first suggested by Kastler in 1950 [12], although it was not made practical until tunable lasers were developed. There are a number of ways in which to apply optical pumping to the cesium-beam standard, but even the simplest methods produce large benefits. Basically, optical state selection is achieved through the frequency selectivity of the exciting light; this laser light is tuned so that atoms in a particular state efficiently absorb the light and are excited to higher electronic states. In relaxing back to the ground state, the excited atoms are restricted by quantum selection rules, so that they can in general relax to only a limited set of the various ground-state levels.

Two types of situations in cesium are particularly interesting. For optical state preparation in NIST-7, atoms are excited from the *F* = 4 ground-state level to the *F* = 3 level of the ^2^P_3/2_ state from whence they can relax to either the *F* = 3 or *F* = 4 level of the ground state. Continued selective pumping from the ground-state *F* = 4 level thus depopulates that level and increases the population in the ground-state *F* = 3 level. Thus, rather than discarding atoms, as is done with the magnetic method, atoms are converted to the desired state. Done with the proper light polarization, this pumping leaves the atoms evenly distributed in population (and velocity) among the seven *m_F_* levels within the *F* = 3 ground state. While a variation on this method using two excitation laser frequencies can achieve conversion of all atoms to the desired *m_F_* = 0 ground-state level, this involves scattering of many more photons into the microwave interrogation region, increasing the light-induced frequency shift commonly called the ac Stark shift.

State detection can also be achieved using optical methods. This is done in NIST-7 by pumping from the *F* = 4 ground state to the *F* = 5 level of the ^2^P_3/2_ state, where quantum selection rules restrict their relaxation to only the *F* = 4 level of the ground state. This is called a cycling transition. Using such a transition, a single atom can be made to scatter a very large number of photons (to fluoresce), thus achieving 100 % detection efficiency. Such a state-detection scheme can readily achieve a detection noise limited by the atom shot noise; that is, the laser-detection process adds no additional noise.

There are several advantages to these optical techniques. First, as just described, for a given flux of atoms, the signal-to-noise ratio is substantially increased, thus decreasing the time needed to make a measurement. Second, the elimination of the state-selection and state-detection magnets removes the troublesome transverse dispersion of atoms associated with the fact that slow and fast atoms in the Maxwellian distribution of atom velocities take different paths through the magnetic optics of such systems. Finally, the asymmetries in the cesium spectrum arising in magnetic state preparation from the velocity selectivity of that process can be essentially eliminated by optical pumping. This means that pulling effects from a line on one side of the central fringe are balanced by an equal line pulling from the other side. These advantages, together with major improvements in the design of the microwave cavity [13] and the servo-control design, have resulted in the achievement of a combined uncertainty for NIST-7 of 4.4×10^−15^, a factor of 20 better than the uncertainty of NBS-6.

**Fig. 2:**
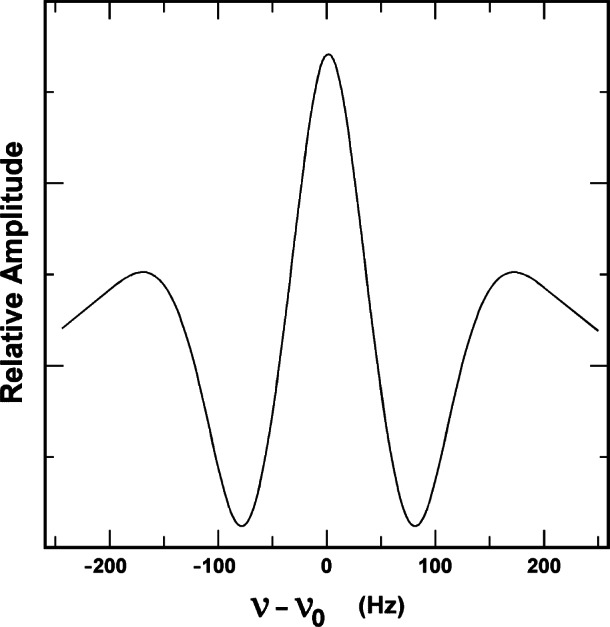
NIST-7 Ramsey pattern (*F* = 3 ↔ *F* = 4) transition in the ground state). The central fringe has a full linewidth at half maximum of about 65 Hz.

The major sources of uncertainty in frequency biases in thermal-beam, cesium frequency standards are second-order Zeeman shift, second-order Doppler shift, end-to-end cavity phase shift and possibly cavity pulling, fluorescence light shift, and line-overlap shift. Because of their sizes, these effects must be considered variable on a level significant to the overall accuracy of the standard. Therefore, they must be evaluated often, and NIST-7 incorporates a number of servo-control systems that allow major portions of this evaluation to be automated. Furthermore, a measurement technique has been developed to allow the very small effects of cavity pulling, line-overlap shift and magnetic-field inhomogeneity to be measured quickly with low-precision measurements [14]. This technique relies on the fact that the shift in the Rabi pedestal part of the line shape is large compared to the shift in the Ramsey fringe part of the line shape. Because of this leverage, the offset between the Ramsey line and its Rabi pedestal must be measured to only about 0.1 Hz to obtain corrections of <1×10^−15^. This requires fractional frequency measurements no better than 1×10^−12^. Combining the digital servo-system and this new measurement technique, the major systematic errors can be evaluated in just a few days. The short-term stability of NIST-7 is typically σ*_y_* (τ) = 7×10^−13^*τ*^−1/2^. This means that frequency measurements with uncertainties near 10^−15^ (one standard deviation) can be made in about 10 hours. Figure 2 shows the Ramsey pattern for the central spectral feature *(F* = 3↔*F* = 4). The linewidth Δ*ν* is about 65 Hz corresponding to Q≡ *ν*_0_/Δ*ν*≈1.5×10^8^. To achieve an uncertainty of 5×10^−15^, the line center must be located with an uncertainty of <1×10^−6^, a difficult task. Furthermore, the very broad velocity distribution found in an optically pumped standard leads to a relatively strong dependence of the frequency on microwave power and modulation parameters. To achieve confidence in measurements to such exacting accuracy, two or more independent techniques have been used to evaluate most of the error sources.

#### 2.2.1 Apparatus

The general layout of this standard is similar to that of the conventional, magnetic-selection standard shown in Fig. 1, except that the state-preparation and state-detection magnets are replaced with laser systems, and the atom detector is replaced with a fluorescence detector. NIST-7 is described in greater detail elsewhere [15,16]. Briefly, it has a Ramsey cavity 1.55 m long and an atomic beam diameter of 3 mm. An axial C field is employed for field uniformity and control of the Rabi-pedestal shape. The cavity ends are designed so that the Poynting vector vanishes at the center of the atomic-beam window [13], thus minimizing distributed-cavity phase-shift effects.

The laser system uses two distributed-Bragg-reflection (DBR) lasers. One is frequency-referenced to the *F* = 4 → *F′* = 5 (*F* refers to a level in the ^2^S_1/2_ state and *F′* refers to a level in the ^2^P_3/2_ state) saturated absorption feature in an evacuated cesium cell. The second laser is frequency-referenced to the *F* = 4 → *F′* = 3 transition.

The digital servo system for locating and locking to the center of the resonance uses a microwave synthesis scheme that involves the addition of a 10.7 MHZ offset near the top of the multiplication chain. This frequency comes from a computer-controlled direct-digital synthesizer (DDS). The entire system is frequency referenced to an active hydrogen maser and the output is in the form of a table of offset values sent to the DDS. This system uses slow, square-wave frequency modulation (≈0.5 Hz) with blanking during the signal transients [17]. Its advantage is its extreme frequency agility that allows interrogation of a number of features in the cesium spectrum.

#### 2.2.2 Magnetic-Field Effects

The C field is operated under closed-loop servo control. This low-duty-cycle (1 %) servo maintains the frequency of the first field-dependent transition to within 10 mHz of a preselected value. This contributes an uncertainty in the second-order Zeeman shift on the clock transition of <1×10^−16^. Field measurements made during assembly [16] showed a fractional field variation at the position of one Ramsey cavity of 5×10^−4^ relative to the mean field. This nonuniformity produces a shift of <1×10^−17^ at all microwave power levels. Measurements of the offsets of the field-dependent Ramsey lines from the centers of their corresponding Rabi pedestals confirm the size of the inhomogeneity shift [14].

#### 2.2.3 Second-Order Doppler Effect

The second-order Doppler shift is of order 3×10^−13^. To achieve the accuracy goal requires a measurement of the effective, ensemble-averaged velocity with an uncertainty of <1 %. This has been done using both a Ramsey lineshape-inversion technique [18] and a pulsed optical-pumping technique [19]. The second-order Doppler correction was computed for several microwave power levels using the two methods. While the corrections varied by nearly 2×10^−13^ over the 7.5 dB power range, the shifts computed from the two methods were in excellent agreement (within 2×10^−15^).

To maintain stability of the second-order Doppler shift to this level requires control of the microwave power experienced by the atoms to much less than 0.1 dB. This is achieved using a power-level servo system with a precision power splitter and a stable power meter. The computer determines the optimum power level through measurement of the signal intensity as a function of microwave power. The Ramsey-inversion program used to measure the velocity profile also returns a measure of the absolute power. The value returned by the Ramsey-inversion program and that determined by the power-level servo system are in excellent agreement.

#### 2.2.4 Cavity-Related Errors

The end-to-end cavity phase shift is measured by reversing the beam direction. The fractional frequency shift on beam reversal is ≈1.2×10^−12^, with an uncertainty of 7×10^−16^. The distributed-cavity phase shift is expected to be small in this machine due to the use of the improved Ramsey cavity [13]. Measurements in both beam directions using beam masks to cover portions of cavity apertures indicate a bias of −1.3×10^−15^ that is corrected to an uncertainty of 4×10^−16^.

Cavity pulling has been investigated by a number of techniques, the most sensitive of which is the measurement of the offset of the Ramsey lines from the centers of their respective Rabi pedestals [14]. The shift under normal operation is −6×10^−15^, and this is corrected to an uncertainty of 5×10^−16^.

#### 2.2.5 Line-Overlap Shift

Because of the symmetry of the spectrum, as well as the very smooth Rabi line wings produced in this standard by the use of an H-plane cavity, Rabi pulling is extremely small. The offset of the Ramsey structures from their corresponding Rabi lines shows that Rabi pulling is <1×10^−16^ for normal operation at a C-field value of 5.4 μT [14].

#### 2.2.6 AC Stark Shift

The ac stark shift caused by the black-body radiation at 39 °C (the operating temperature of the beam tube) is calculated to be 2.0×10^−14^ [20]. It is too small to be measured by actual temperature change in the standard but must be accounted for in the evaluation. The uncertainty in this value is <5×10^−16^.

An ac stark shift is also possible from the near resonant light that is a byproduct of the optical pumping process (fluorescence). Theoretical analysis of this effect in the geometry of NIST-7 shows it to be negligible at the 10^−15^ level [21]. Measurement of this effect shows it to be small, but the uncertainty in this measurement is 3×10^−15^, making it the largest systematic error for the standard. Further improvements in the measurement method should reduce this uncertainty.

#### 2.2.7 Microwave Leakage

This effect arises from stray microwave radiation in the vicinity of the Ramsey cavity. It has the same effect as end-to-end phase shift, and would be accounted for in a beam reversal if it were stable in both phase and amplitude at every point in space and time. Leakage from microwave structures outside the beam tube that finds its way into the standard is not stable in phase and amplitude because it travels over uncontrolled and varying pathways. It contributes to both frequency errors and long-term instability in the standard. A heterodyne detector was used to locate the sources of this external leakage and an antenna was used to probe the points where the radiation couples to the standard [22]. Measures were taken to substantially reduce both the sources and the coupling. The shift produced by this external radiation is now <1×10^−16^.

#### 2.2.8 Electronics

The digital servo system is susceptible to errors from modulation distortion and integrator offsets. The servo can also have subtle errors from switching transients, round-off errors and aliasing. These can be affected by rf spectral purity and phase noise. Rather than use the frequency of the standard as a diagnostic tool to study these effects, a painfully slow process, quick and sensitive electronic tests have been used. Models for the sensitivity to all superpositions of amplitude modulation (AM) and phase modulation (PM) noise and methods for measuring these effects have been developed [17]. The total error found to arise in these electronic systems is <1×10^−15^.

#### 2.2.9 Summary of Performance of NIST-7

The sources of major frequency bias are independently evaluated with just a few days of measurements. Complete evaluation of all other small effects takes much longer, but such effects are stable at the 10^−15^ level and their infrequent evaluation does not detract from the uncertainty of the standard. Table 1 lists all known frequency shifts and the uncertainties associated with their correction. The combined uncertainty of the standard is taken to be the square root of the sum of the squares of these uncertainties. For the most recent evaluation represented by this table, the combined uncertainty is 4.4×10^−15^.

## 3. Cesium-Fountain Frequency Standard

The fountain concept for extending atom observation time was introduced by Zacharias in 1954 [23]. Laser cooling of atoms was unknown at the time, but Zacharias believed that it might be possible to direct a thermal beam of atoms upward and then depend on finding that a small number of slower atoms in the Maxwellian velocity distribution would reach apogee within the device and return to the source. While there would be a dramatic loss of signal, the time of flight for atoms going up 1 m and returning would be on the order of 1 s, resulting in a large reduction in resonance linewidth. Furthermore, atoms could traverse the same microwave cavity twice (once on the way up and once on the way down), and this would provide for Ramsey interrogation (temporally separated rather than spatially separated regions) without the end-to-end cavity phase shift found in beam standards. The experiment was attempted shortly after the proposal was made, but no return signal was observed. It was later found that scattering processes within the beam have the effect of removing the slowest of atoms, so it is clear now that the original experiment was doomed to failure. The concept was not to be proven until 35 years later, after laser cooling techniques were developed.

**Fig. 3:**
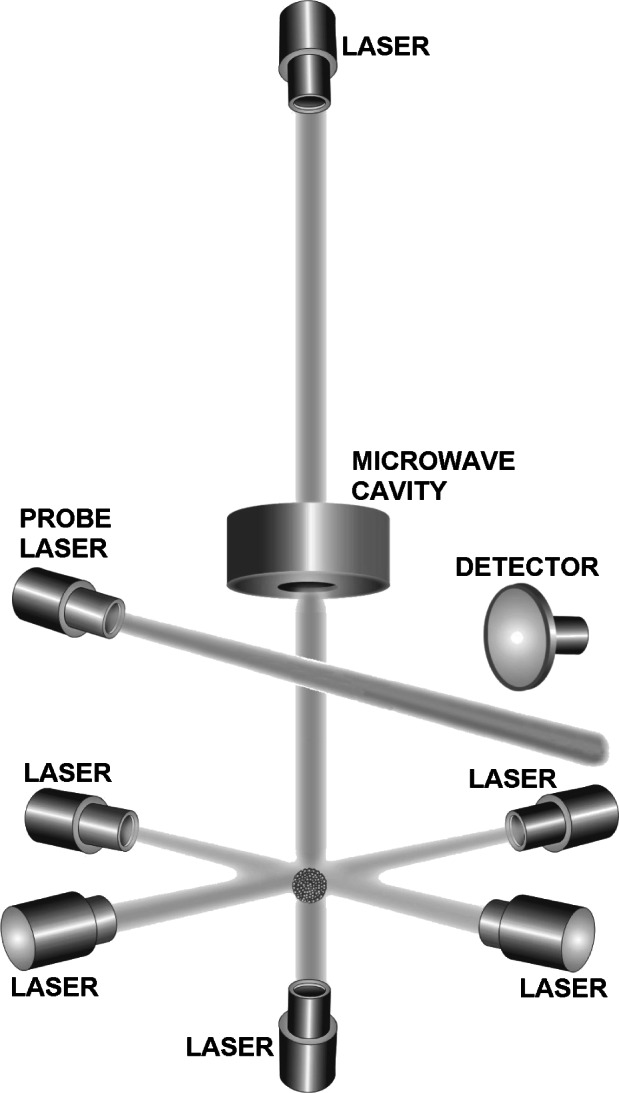
Fountain concept. Atoms are trapped at the intersection of the six orthogonal laser beams and are tossed vertically by slightly offsetting the frequencies of the two vertical lasers and then turning all six lasers off. The atoms rise and fall through the microwave cavity and undergo state interrogation below the microwave cavity.

The first demonstration of the fountain concept was at Stanford University [24] in 1989 and the first primary standard based on the fountain concept was developed shortly thereafter by a group at the Laboratoire Primaire du Temps et Frιquences (LPTF) [25]. The laser-cooled fountain concept is shown in Fig. 3. The atom ball in the LPTF cesium fountain is formed either using a magneto-optical trap (MOT) or optical-molasses [26]. The NIST fountain collects atoms only in optical molasses. The MOT can achieve higher atom density, but the ball of atoms is typically converted to optical molasses prior to launching. For fountain frequency standards the transverse temperature of the atoms is a key parameter. During flight of the atoms through the devices, a large fraction (of order 90 %) of the atoms with higher transverse velocities are lost before they return to the detection region. Additional transverse cooling would allow increased utilization of source atoms, and a better trade-off between signal strength and spin-exchange shift.

### 3.1 Description of NIST-F1

The NIST cesium fountain is described in greater detail elsewhere [27] and only a short overview is given here. The NIST-F1 optical-molasses source gathers approximately 10^7^ cesium atoms at <2 μK in about 0.4 s. The ball of atoms is then launched by differential detuning of the two vertical laser beams to make a moving optical molasses. After the atoms have been accelerated to their launch velocity the molasses laser beams are all detuned to the red in frequency while simultaneously reducing the optical intensity to further cool the launched atom sample.

The atoms travel from the optical molasses source region through a region that is used to detect atoms later in the process, and into the magnetically shielded C-field section of the fountain. All of the launched atoms at this point are in the *F* = 4 state and approximately evenly distributed over all possible *m_F_*-state values. The atoms first encounter a microwave state-preparation cavity, which moves the |4,0〉 atoms to the |3,0〉 state using a π-pulse at 9.192 GHz. Any remaining *F* = 4 atoms are then removed from the sample with an optical pulse.

The remaining atoms in the |3,0〉 state next encounter the Ramsey microwave cavity, where the microwave field prepares the atoms in a superposition state of |4,0〉 and |3,0〉. The Ramsey cavity, a TE_011_ OFHC copper cavity, has been previously described [28]. After the Ramsey cavity the atoms drift upward in the flight-tube, achieve apogee, then move downward under the influence of gravity, and re-enter the Ramsey cavity where the Ramsey-interrogation process is completed. After leaving the Ramsey cavity the atoms next fall through the state-preparation cavity, in which the microwave drive has been both detuned by 12 MHZ and attenuated by more than 100 dB, to prevent unwanted interactions with this field.

Finally, upon exiting the C-field region, the atoms enter the detection region. Here atoms in the |4,0〉 state are first detected by fluorescence in an optical standing wave and then removed from the atomic sample by an optical traveling wave. The sample then traverses an optical re-pump beam which transfers |3,0〉 atoms to the |4,0〉 state. These |4,0〉 atoms (formerly |3,0〉 atoms) are then detected by optical fluorescence in a standing wave similar to the one described above. The point of detecting both the |4,0〉 and |3,0〉 atoms is to form the sum of the two, a number which is proportional to the number of atoms launched. This sum can be used to normalize the number of atoms in each ball, thus removing noise that would arise from toss-to-toss, atom-number fluctuations.

**Fig. 4:**
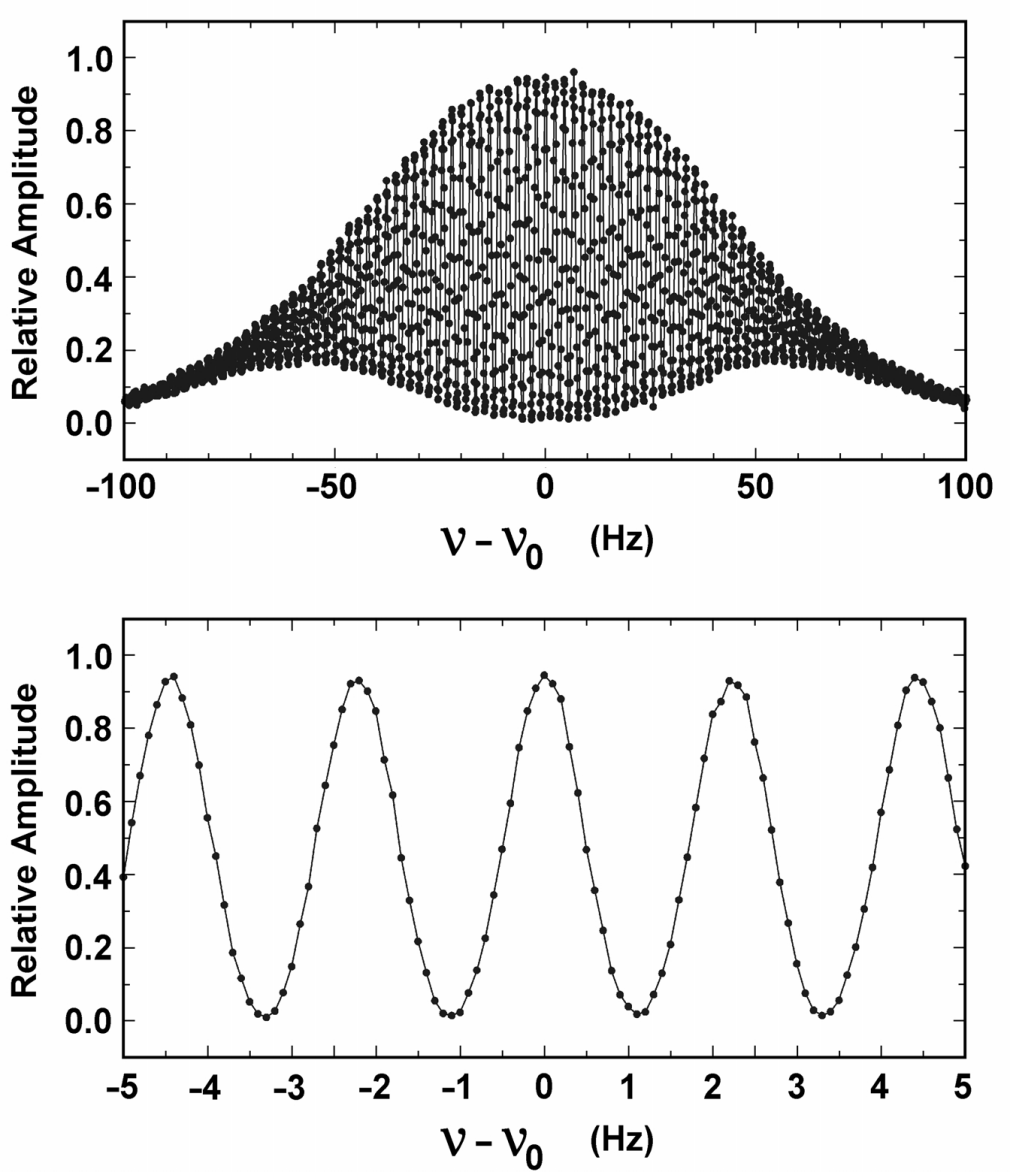
NIST-F1 Ramsey pattern. The upper portion of the figure shows the entire ground state (*F* = 3 ↔ *F* = 4) pattern, while the lower portion of the figure shows an expanded view of the central fringe. The full linewidth at half maximum is about 1 Hz.

Figure 4 shows the Ramsey pattern for NIST-F1. The upper portion of the figure shows the envelope of the pattern and the lower portion shows an expanded section around the central Ramsey fringe. The large number of fringes is a consequence of the narrower velocity distribution. The linewidth of the central fringe is ≈1 Hz corresponding to a *Q* ≡ *ν*_0_/Δ*ν* ≈ 10^10^. One side of the Ramsey fringe is probed in a cycle involving the launch of a ball of atoms, microwave interrogation, and then optical detection. The microwave synthesizer is then tuned to the other side of the fringe and the cycle is repeated. A single measurement of the cesium clock frequency consists of two measurement cycles, one on each side of the Ramsey fringe. The servo-control systems acts to equalize the signal measured on each side of the fringe, thus assuring that line center is at the midpoint of the two frequencies.

### 3.2 Systematic Frequency Biases

Because of the symmetry of the fountain geometry, the low velocities of the atoms, and the narrow linewidth, many frequency shifts are much smaller than those found in thermal-beam standards. A number of systematic effects have been shown to have a worst-case frequency bias Δ*ν*/*ν*_0_ of 10^−16^ or less in this fountain. These effects, which will not be discussed further, are cavity pulling, distributed-cavity phase shift (first-order Doppler shift), Rabi pulling, Ramsey pulling, second-order Doppler shift, dc Stark shift, and the Bloch-Siegert shift.

#### 3.2.1 Second-Order Zeeman Shift

The C field used in NIST-F1 is about 0.1 μT (1 mG) and causes a 5×10^−14^ fractional frequency shift due to the second-order Zeeman effect. This shift is evaluated by measuring the frequency of the |4,1〉→|3,1〉 magnetic-field-sensitive transition and using the frequency of that transition to correct for the shift in the |4,0〉→|3,0〉 transition. To sufficient accuracy the fractional Zeeman correction is then given by

$$\frac{\delta \nu_{z}}{\nu_{0}}\approx \frac{8\delta \nu_{1,0}^{2}}{\nu_{0}^{2}},$$ (2)

where *δν*_1,0_ is the measured difference frequency between the |4,0〉→|3,0〉 and the |4,1〉→|3,1〉 transitions. Several things must be considered to achieve accuracy in the evaluation of this shift.

Due to the large number of Ramsey fringes, there is an uncertainty in identifying the central fringe on the magnetically sensitive transitions. A magnetic-field inhomogeneity will shift the Ramsey fringes with respect to the underlying Rabi pedestal. The central fringe is identified in two ways. First, a magnetic-field map is constructed by launching the atoms to various heights and applying a Rabi pulse on a magnetically sensitive transition at apogee using an antenna in the drift region. This technique is described by the LPTF group [25]. A better method uses repeated measurements of the Ramsey fringe pattern around the central fringe for the *m_F_* = 1 transition while launching to various heights. The peaks of the Ramsey fringes constructively interfere on the central fringe and lose coherence away from it. A mis-assignment of even 1 full fringe (considered unlikely) would produce a fractional frequency error of only about 3×10^−16^.

The stability of NIST-F1 when locked to the |4,1〉→|3,1〉 line shows a flicker-noise floor at *σ_y_*(*τ*) = 10^−12^, which indicates that magnetic-field fluctuations of about 10^−12^ T are present inside the C-field region. Field fluctuations of this size cause a frequency shift in the *m_F_* = 0 clock transition of order 10^−18^ and are ignored here.

Measurement of the |4,1〉→|3,1〉 transition determines the temporal average of the magnetic field *B* over the flight time. However, the temporal average of *B*^2^ is needed to correct the second-order Zeeman shift. If the magnetic field is modeled as seen by the atoms as *H*(*t*) = *H*_0_[1−*εf*(*t*) where *f*(*t*) is a function with |*f*(*t*)|≤1, and *ε* is a scaling factor, then the difference between the mean square and the square of the mean leads to a frequency shift given by

$$\frac{\Delta \nu }{\nu_{0}}=\frac{(427\times 10^{8}H_{0}^{2})\varepsilon^{2}}{\nu_{0}}[{\langle f(t)\rangle }^{2}-\langle f{\left(t\right)}^{2}\rangle ]$$ (3)

From the magnetic field model, *ε* can be shown to be of order 0.1, and from consideration of atom ballistics, 〈*f*(*t*)^2^〉−〈*f*(*t*)^2^〉 is found to be ≤0.01. The maximum inhomogeneity frequency shift is then less than Δ*ν*/*ν*_0_ = 10^−17^.

The uncertainty associated with the quadratic Zeeman shift is therefore dominated by problems associated with location of the central fringe and is conservatively assigned a value equivalent to the mis-assignment of one whole fringe in the *m_F_* = 1 manifold, that is, 3×10^−16^.

#### 3.2.2 Spin-Exchange Frequency Shift

The evaluation of the spin-exchange frequency shift requires a measurement of the atomic density, which is determined using several methods. This involves carefully calibrating the entire detection system including the size of the detection beams and their intensity, the solid angle for collection of photons from the atomic sample, and finally a calibration of the photodiode and its associated amplifier. The average density is determined from a measurement of the number of atoms launched by using the detection region to measure the number of atoms in the cloud on the way up and again on the way down. Assuming Maxwellian thermal distributions and extracting the physical dimensions of the launched atom ball from the data, the atomic density can be determined as a function of time. This density is then used, along with a spin-exchange frequency-shift coefficient for the *m_F_* = 0 state as measured by the LPTF group [29] and the Stanford group [30] to determine the total spin-exchange frequency shift. The process is complicated by the use of a pure molasses source, since the initial cloud size is uncertain and this propagates into the final result both directly and as an uncertainty in the atom temperature. The atomic density derived above is next used to predict the total spin-exchange frequency shift as

$$\frac{\Delta \nu }{\nu_{0}}=\left(-2\times 10^{-21}\right)\left[\left(\frac{ht}{T}\right)\left(\frac{\rho_{1}+\rho_{3}}{2}-\bar{\rho_{2}}\right)+\bar{\rho_{2}}\right]$$ (4)

where *h* is a factor of order unity and depends on the excitation power, *t* is the Rabi time, *T* is the Ramsey time, $\bar{\rho_{1}}$ and $\bar{\rho_{3}}$ are the atomic density on the first and second pass through the microwave cavity, respectively, and $\bar{\rho_{2}}$ is the average density over the entire Ramsey time. This formula, originally derived by Shirley [31] for Zeeman shifts over the length of a beam tube, has been modified for the case at hand.

As a consistency check the frequency of the fountain with state selection is compared to the frequency with no state selection. Without state selection the density is much higher, although the spin-exchange cross section is smaller by a factor of two. No spin-exchange frequency shift is seen at 3×10^−15^ (limited by the random uncertainty of the measurement) when this test is made. The total calculated spin-exchange shift in the fountain is typically 5×10^−16^ and as a result of the difficulties associated with the density determination mentioned above, a conservative uncertainty of 5×10^−16^ is assigned. A rigorous analytic solution to the density as a function of time is needed. This will allow further improvement in evaluation of the spin-exchange uncertainty.

#### 3.2.3 Blackbody Radiation Shift

The next significant systematic frequency bias is the blackbody radiation shift. This radiation shift is the same as that described for NIST-7, but it is somewhat more significant, because the overall uncertainty for this standard is lower. The cavity and drift tube region of the fountain are temperature controlled at a temperature of 41 °C. Sensors on the microwave cavities and the drift tube of the fountain monitor the temperature. The largest temperature differential noted from these sensors is 0.2 °C. A temperature error of 1 °C is taken to be the worst possible error and this leads to an uncertainty of less than 3×10^−16^.

#### 3.2.4 Gravitational Redshift

The gravitational frequency shift (redshift) in Boulder is large, about −1.8×10^−13^. The gravitational potential in Boulder Colorado relative to the geoid has been reevaluated using an Earth potential model and the resulting claimed uncertainty on the frequency correction is less than 5×10^−16^.

#### 3.2.5 Summary of Uncertainty

The present values of the most-significant systematic frequency biases in NIST-F1 are given in Table 2. The overall Type B uncertainty (systematic effects) is 0.8×10^−15^, dominated by the spin-exchange shift. The Type A (statistical) uncertainty is 1.3×10^−15^, resulting in a combined uncertainty of 1.5×10^−15^. This represents the present status of the standard. Work during the next year should reduce the combined uncertainty to <1×10^−15^. However, because of the spin-exchange shift, it will be difficult to achieve an uncertainty much below 5×10^−16^.

## 4. Stored-Ion Frequency Standards

The key advantage of using stored ions for frequency standards is that they can be stored for long periods (hours to days and even weeks are common) with, in some cases, exceedingly small systematic frequency shifts. This allows for an arbitrarily large increase in observation time over the cesium-beam and atomic-fountain methods and results in very narrow linewidths Δ*ν*/*ν*_0_. Ramsey interrogation is accomplished by subjecting the ions to pairs of microwaves pulses, and the linewidth is then inversely proportional to the time interval between the pulses. The end-to-end cavity phase shift of cesium-beam standards is absent, and there is no first-order Doppler shift.

The first stored-ion frequency standard that exhibited a reasonably small uncertainty (1×10^−13^) was a Be^+^ ion standard operating at 303 MHz [32]. While this standard used a modest ion cloud (≈10^4^ ions), the standards described below use only a few ions. Despite the small number of ions in these standards, very competitive stabilities have been achieved for the microwave-frequency standard, and the short-term stability achieved in experiments on the optical-frequency standard is exceptionally high. Storage methods [33] include both radio-frequency traps (called Paul traps), which use a combination of static and ac electric fields to achieve confinement, and Penning traps, which confine ions using a combination of static electric and magnetic fields. There are a variety of ions and trap configurations that have been used, but this discussion is limited to work done at NIST on ^199^Hg^+^ ions stored in a linear rf trap to produce a microwave frequency standard and in a similar linear trap to produce an optical frequency standard.

**Fig. 5:**
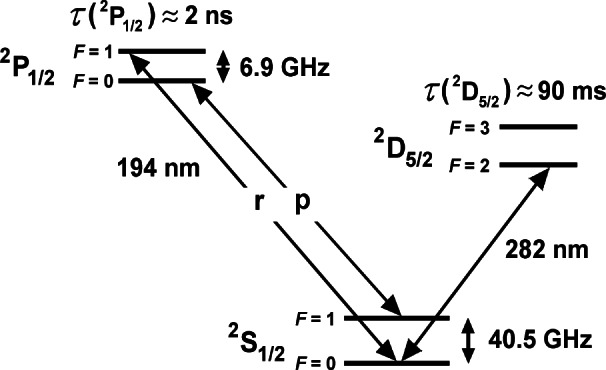
Partial energy diagram of ^199^Hg^+^.

^199^Hg^+^ offers a microwave clock transition at 40.5 GHz and an optical clock transition at 1.06×10^15^ Hz. (see Fig. 5). To first order, both transitions are insensitive to uniform magnetic fields. Using a linear trap, uncertainties in all systematic frequency shifts of the 40.5 GHz transition are expected to be less than 1×10^−16^. Using a single ion, the relative uncertainty is expected to be <1×10^−17^ for the optical transition.

If fluctuations of the atomic signal are due only to quantum statistics, then the stability of a frequency source servoed to the atomic transition is given by [34]

$$\sigma_{y}(\tau )=\frac{2\pi }{\nu_{0}\sqrt{NT_{R}}}\tau^{-1/2},$$ (5)

where *ν*_0_ is the frequency of the atomic transition, *N* is the number of ions, *T*_R_ is the Ramsey interrogation time, and *τ* is the averaging time of the measurement. For the ground-state hyperfine transition, *ν*_0_ = 40.5 GHz. It appears feasible to use *N* = 100 ions and *T*_R_ = 100 s, which gives *σ_y_*(*τ*) ≈ 4×10^−14^*τ*^−1/2^. For the 282 nm ^2^S_1/2_ → ^2^D_5/2_ electric quadrupole transition, *ν*_0_ ≈ 10^15^ Hz, so that using *N* = 1 and *T*_R_ = 25 ms gives *σ_y_*(*τ*) ≈ 10^−15^*τ*^−1/2^.

### 4.1 Cryogenic Linear RF Trap

**Fig. 6:**
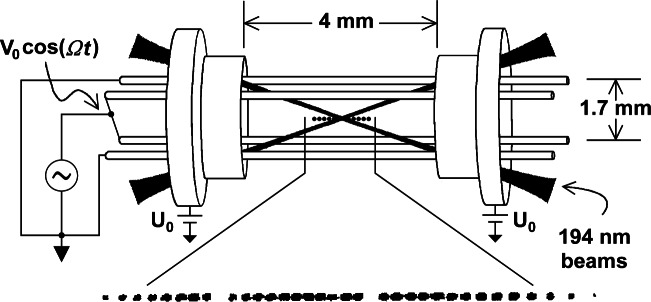
Linear rf trap, and an image of a linear ion crystal. The ions are spaced approximately 10 μm apart, and the gaps in the ion crystal are due to ions other than ^199^Hg^+^ that do not fluoresce at the frequencies of the 194 nm laser beams. The spatial extent of the ions is exaggerated for clarity; in reality the laser beams simultaneously overlap all the ions.

Figure 6 shows the linear rf trap [35] used in the 40.5 GHz microwave frequency standard. Two diagonally opposite rods are grounded, while the potential of the other two rods is *V*_0_ cos(*Ωt*), where nominally *V*_0_ ≈ 150 V and *Ω*/2*π* = 8.6 MHZ. The resulting pseudopotential confines the ions radially in a well with a secular frequency *ν*_r_ ≈ 230 kHz. To reduce patch fields and remove electrical charge that otherwise leaves the rods slowly in the cryogenic environment, resistive wires are threaded through the rods to heat them during and after loading the trap. Two cylindrical sections at either end of the trap are held at a potential of approximately +10 V, confining the ions axially. The ions are laser-cooled using the 194 nm ^2^S_1/2_ → ^2^P_1/2_ electric-dipole transitions shown in Fig. 5. Typically, a string of approximately ten ions is confined near the trap axis. By minimizing the ion micromotion in all three dimensions, the laser-cooled ions are made to lie along the rf nodal line [36]. Here, parametric heating is essentially eliminated, so the cooling-laser radiation, which perturbs the clock states, can be removed during the long probe periods of the clock transition.

The trap is placed in a liquid-helium (4 K) cryogenic environment in which Hg and most background gases are cryopumped onto the walls of the chamber. This essentially eliminates ion loss due to collisions with the background gas. In addition, collision shifts should be negligible. Operation at 4 K also reduces the frequency shifts due to blackbody radiation of the ^199^Hg^+^ ground-state hyperfine transition. At *T* = 4 K, the fractional blackbody Zeeman shift is −2×10^−21^, and the fractional blackbody Stark shift is −3×10^−24^ [20].

#### 4.1.2 Laser-Atom Interactions

Laser beams at 194 nm [37] are used for cooling, state preparation, and determining the internal states of the ions. To cool the ions, the frequency of a primary laser is tuned slightly below resonance with transition p (see Fig. 5). Although this is a cycling transition, the laser can off-resonantly excite an ion into the ^2^P_1/2_, *F* = 1 level, from which the ion can decay into the ^2^S_1/2_, *F* = 0 level. To maintain fluorescence, a repumping laser beam, resonant with transition r in Fig. 5, is overlapped collinearly with the primary laser beam. To prevent optical pumping into the dark states of the ground state *F* = 1 level, the 194 nm radiation (containing both r and p components) is split into two beams that are made to propagate through the trap at an angle of 40° relative to each other (±20° relative to the trap axis as shown in Fig. 6). The polarization of one beam is in the plane of the 194 nm beams, while the polarization of the other 194 nm beam is continuously modulated between left and right circular.

#### 4.1.3 Operation of the 40.5 GHz Microwave Clock

The ions are prepared in the ^2^S_1/2_, *F* = 0 state by turning the repumping beam off. The atomic state after the clock radiation is applied is determined by pulsing on only the primary beam p for a time comparable to the time necessary to pump the ions from the ^2^S_1/2_, *F* = 1 to the ^2^S_1/2_, *F* = 0 level (typically 10 ms). If the ion is found in the ^2^S_1/2_, *F* = 1 level, it will scatter about 10^4^ photons before it optically pumps into the 2S_1/2_, *F* = 0 level. Approximately 150 of these photons are detected and counted. If the ion is found in the ^2^S_1/2_, *F* = 0 level, it will scatter only a few photons.

For the first part of the measurement cycle, the ions are cooled by pulsing on both the primary and repumping 194 nm laser beams for 300 ms. Next, the repumping beam is turned off for about 60 ms, so that essentially all of the ions are optically pumped into the ^2^S_1/2_, *F* = 0 level. The clock transition is probed using the Ramsey technique of separated oscillatory fields [11]. Both beams are blocked during the Ramsey microwave interrogation period, which consists of two 250 ms microwave pulses separated by the free precession period *T*_R_. *T*_R_ is varied from 2 s to 200 s. Finally, the primary beam is turned on for 10 ms to 20 ms while counting the number of detected photons. This determines the ensemble average of the atomic state population and completes one measurement cycle.

The microwave frequency is derived from a synthesizer locked to a hydrogen maser. Stepping the synthesizer frequency between measurement cycles produces a set of Ramsey fringes. A digital servo locks the average synthesizer frequency to the central fringe. For seven trapped ions and *T*_R_ = 100 s, the fractional frequency stability of the servoed oscillator is *σ_y_* (*τ*) = 3×10^−13^*τ*^−1/2^ for 2 h. Consistently, the measured *σ_y_*(*τ*) is about twice the value expected from Eq. (1), primarily because of laser intensity fluctuations at the site of the ions. The stability of the ion standard is comparable to the stabilities of NIST-7 and NIST-F1.

#### 4.1.4 Measurement Uncertainties

Table 3 shows the most important corrections made to the average frequency for each run [38]. The fractional Zeeman shift due to the static magnetic flux density is +0.24 *B*_static_^2^, where *B*_static_ is in tesla. The measured Zeeman splitting of the ground state transitions gives *B*_static_ ≈ 3×10^−7^ T, with fluctuations of 1×10^−8^ T. Thus the fractional uncertainty in this Zeeman shift is 1.4×10^−15^.

A correction is also made for the ac Zeeman shift that depends linearly on the rf power delivered to the trap. This shift is caused by magnetic fields due to asymmetric currents at frequency *Ω*/2*π* in the trap electrodes. These currents are caused by capacitance between the electrodes and stray capacitance to the ground plane.

Considering the possibility that the current distribution may vary from load to load, the average transition frequency versus rf power is measured for each ion crystal, and in each case this is extrapolated to zero shift to give the unshifted frequency *ν*_0_. An average was taken over five different ion crystals and *ν*_0_ was found to be independent of time within the uncertainty of the measurement. The uncertainty in the extrapolated frequency averaged over the five different crystals used in the frequency measurement is 3.2×10^−15^.

The magnitudes of several frequency shifts scale with the free precession time as 1/*T*_R_. These include shifts due to the phase chirp of the microwaves as they are switched on and off, any leakage microwaves as they are switched on and off, any leakage microwave field present during the free precession time *T*_R_, and asymmetries in the microwave spectrum. By varying *T*_R_, the fractional shift from these combined effects is measured to be −3(3)×10^−14^/*T*_R_ (where *T*_R_ is in s). The parenthetical 3 is the uncertainty of this measurement.

The frequency of the reference maser is determined by comparing it to the frequency of International Atomic Time (TAI). This determines the average frequency of the Hg^+^ standard to be *ν*_0_ = 40 507 347 996.841 59(14)(41) Hz. Here, the first uncertainty is due to the systematic shifts shown in Table 3. The second uncertainty is due to the quoted uncertainty in the frequency of TAI. The Type B (systematic) uncertainty (3.4×10^−15^) is roughly comparable to the results obtained with cesium-beam and cesium-fountain frequency standards.

The main uncertainties will certainly be reduced in future work. Better magnetic shielding will reduce fluctuations in the static magnetic field. The magnetic field at frequency *Ω*/2*π* can be reduced by lowering *ω*_r_ and the trap dimensions. Finally, by monitoring each ion individually, the internal states can be determined with negligible uncertainty, which will eliminate noise due to frequency and intensity fluctuations of the detection laser.

### 4.2 Optical Frequency Standard

A frequency standard based on the ^199^Hg^+^, 282 nm electric quadrupole transition (^2^S_1/2_↔^2^D_5/2_ transition shown in Fig. 5), which has a natural linewidth of 1/(2*πτ*_D_) = 1.7 Hz (where *τ*_D_ = 90 ms is the lifetime of the ^2^D_5/2_ state), is now being investigated [39]. Since the clock transition *ν*_0_ is in the optical region (*ν*_0_ = 1.06×10^15^ Hz), the fractional frequency stability can be very high as seen from Eq. (5). For example, for a single ion probed using the Ramsey technique with a free precession time of 25 ms, the fractional frequency stability is about 1×10^−15^*τ*^−1/2^, two orders of magnitude lower than that of the Hg^+^ microwave standard described above.

**Fig. 7:**
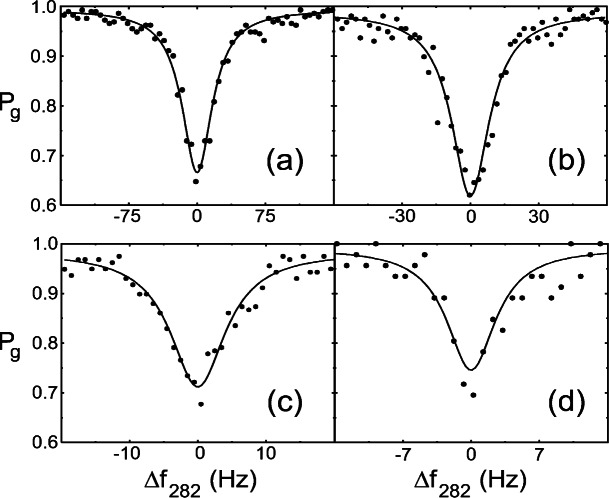
Absorption spectra of the ^2^S_1/2_ (*F* = 0) ↔ ^2^*D*_5/2_ (*F* = 2) *m_F_* = 0 electric quadrupole transition in ^199^Hg^+^. Δ*f*_282_ is the frequency of the 282 nm probe laser detuning and *P_g_* is the probability of finding the atom in the ground state following state interrogation. The plots show four different lengths of excitation: (a) 20 ms (averaged over 292 sweeps), (b) 40 ms (158 sweeps), (c) 80 ms (158 sweeps), and (d) 120 ms (46 sweeps).

The frequency of a stabilized laser at 563 nm has been doubled and then locked to this transition in a single ion confined near the Lamb-Dicke limit in a linear Paul trap [40]. The laser linewidth probing this transition has been independently measured to be as low as 0.22 Hz for a measurement time of 20 s [41]. Figure 7 shows the linewidth of the transition for various lengths of the excitation pulse. The narrowest Fourier-transform-limited linewidth Δ*ν* of 6.7 Hz is obtained for a pulse time of 120 ms (33 % longer than the D-state lifetime!). This corresponds to *Q*≡ *ν*_0_/Δ*ν* ≈ 1.6×10^14^, the largest *Q* ever achieved for optical spectroscopy. This high *Q*, combined with the relative freedom from environmental perturbation afforded by ion trapping, suggest that trapped-ion optical-frequency standards should have significant advantages over present microwave frequency standards.

These experiments used a cryogenically cooled, linear quadrupole trap similar to that used for the microwave standard described in the previous section. A single ion can be trapped and contained for an arbitrary period in this trap (a single ion was held for several months until it was deliberately removed). The background gas pressure in the cryogenic trap is low, and there appear to be no observable frequency shifts associated with any residual background-gas interactions. The present system is not magnetically shielded, and both magnetic-field shifts and electric-field shifts arising from patch potentials have been identified and evaluated at a level of 1×10^−15^. It should be possible to reduce the uncertainties associated with all such effects to values approaching 1×10^−18^, particularly if the linear quadrupole trap is replaced by a spherical rf quadrupole trap that does not rely on static electric potentials for confinement. While this optical electric-quadrupole transition in ^199^Hg^+^ ion shows significant promise as a frequency standard, still narrower linewidth and lower uncertainty might be more easily achieved using other transitions (such as electric-dipole transitions) in other elements.

Optical frequency standards, which enjoy a large advantage because of their very high *Q*, have not previously been favored as primary standards, since most applications of frequency standards are in the rf/microwave region of the spectrum, and it was difficult to accurately relate optical and microwave frequencies. However, recently developed methods for accurately measuring optical frequencies [42, 43] appear promising, and optical frequency standards should start to challenge their microwave counterparts in the next few years.

## 5. Summary and Discussion

**Fig. 8:**
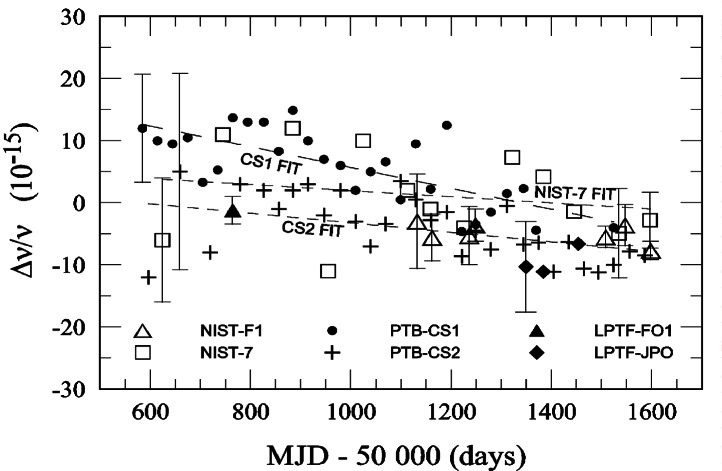
Combined uncertainties of NIST-7 and NIST-F1 compared to combined uncertainties of several other national primary frequency standards through NIST AT1E, a special post-processed time scale with exceptional long-term stability. Data on the French standards (designated LPTF) and German standards (designated PTB) are obtained from publications of the Bureau International des Poids et Mesures. MJD is the modified Julian date (the Julian date minus 2 400 000.5 d). The uncertainty bars represent one standard adeviation estimates.

Figure 8 compares the uncertainties of a number of primary frequency standards, including NIST-7 and NIST-F1, for a period of more than 1000 days. This comparison involves evaluating the fractional frequency offset between a stable (but not accurate) frequency reference, AT1E, and six of the world’s best primary frequency standards over a period of more than 1000 days. AT1E is a post-processed ensemble of five hydrogen masers at NIST and has a stability better than 1×10^−15^ for time intervals up to 100 days [44]. This comparison could also have been made using International Atomic Time (TAI), except that the short-term stability of TAI is not as good as that of AT1E due to the noise of the time-transfer process involved in generating TAI. Some of the NIST-F1 measurements were made over short time intervals (on the order of a few days) and the noise of TAI would have dominated.

In addition to NIST-F1 and NIST-7, Fig. 8 also shows data for two standards from Physikalisch-Technische Bundesanstalt (PTB) in Germany and two from LPTF in France. The PTB standards are both magnetically state-selected thermal-beam standards, while the LPTF standards are a fountain standard (LPTF-F01) and an optically pumped thermal-beam standard (LPTF-JPO). Representative uncertainties for each standard are shown by the uncertainty bars. The fitted lines are calculated by the linear least-squares method and help to illustrate the long-term trends. AT1E exhibits a slow downward frequency drift, but the relative frequency offsets and frequency drifts of the standards are real. Though there are a few points that are outside the uncertainty bars, the agreement among the various standards has generally been very good.

The NIST cesium-beam primary frequency standard has been engineered to run routinely. Substantially less engineering development has been done on the cesium-fountain frequency standard, which went into service only recently, and very little has been done to engineer a stored-ion frequency standard that can run routinely, primarily because the concepts for ion frequency standards have been evolving so rapidly. However, because of the narrow linewidths that can be realized and the very small values for systematic frequency shifts, the performance of the stored-ion standards likely will surpass those of the neutral-atom devices. Optical transitions in ^199^Hg^+^ or other ions are particularly interesting, because the relative linewidth (1/*Q*) is exceedingly small, systematic frequency shifts appear to be substantially smaller than those of neutral atom standards, and very high stability can be readily achieved. The recent demonstration of relatively simple optical combs that allow an accurate connection between the microwave and visible regions has removed a significant barrier to the use of optical transitions for primary frequency standards, and should speed progress toward the development of an optical frequency and time standard.

**Fig. 9:**
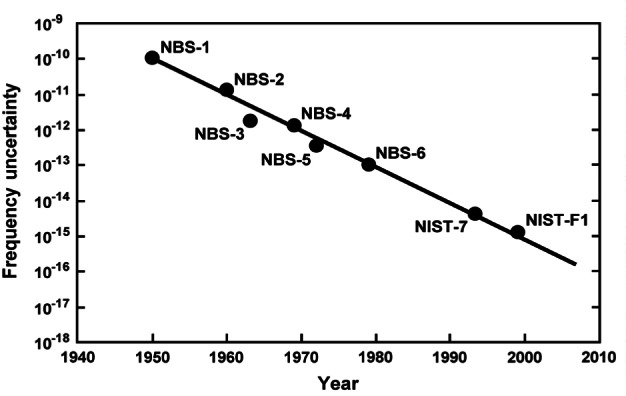
Uncertainties of NBS/NIST standards since 1950.

It is interesting to consider the steady progress in improving frequency standards over the last fifty years. The historical record of the uncertainties of NBS/NIST atomic frequency standards is shown in Fig. 9. The roughly linear fit through these points indicates a reduction in uncertainty of better than one order of magnitude per decade. The development of primary frequency standards has been greatly stimulated by the new laser-cooling and state-control methods.

In conclusion, it is worth noting that the first cooling below ambient temperature of any atomic species was done at NIST in 1978 [45] and the first neutral-atom laser cooling was also demonstrated at NIST in 1982 [46]. These methods were developed just as the cesium-beam frequency standards were reaching their natural limits of evolution. Further improvement in these standards would have been small and difficult to achieve, since the key systematic frequency shifts limiting their performance were directly related to the larger (thermal) velocities of the atoms. For the moment it appears that the new methods can provide improvements of perhaps another two orders of magnitude or more, but many practical problems must be addressed in the process.

## Figures and Tables

**Table 1:** Summary of frequency shifts and their Type B uncertainties in NIST-7. The total Type B uncertainty is 4.1×10^−15^

Physical effect	Bias (×10^−15^)	Type B uncertainty (×10^−15^)
Second-order Doppler		
West-to-east	−288	1
East-to-west	−287	
Second-order Zeeman	+147 676.0	0.1
Cavity pulling	−6.0	0.5
Rabi pulling	< −0.1	0.1
Cavity phase (end-to-end)		
West-to-east	+717	0.1
East-to-west	−617	
Cavity phase (distributed)	−1.3	0.4
Fluorescence light	−0.01	3
Blackbody	−20.4	0.5
Gravitation	+179.9	0.1

Electronics		

rf spectral purity	0	0.1
Integrator offset	0	1
AM on rf or laser	0	1
Microwave leakage	0	0.1
PLL and optical transients	−1.5	2

**Table 2:** Summary of the largest systematic frequency shifts in NIST-F1. The uncertainties for all items not listed are <10^−16^. The total type B uncertainty is 0.8×10^−15^

Physical effect	Bias (×10^−15^)	Type B uncertainty (×10^−15^)
Second-order Zeeman	+45.0	0.3
Spin exchange	−0.9	0.5
Blackbody	−20.6	0.3
Gravitation	+180.54	0.1
Microwave leakage	0	0.2

**Table 3:** Largest systematic shifts of the average mercury-ion-clock transition frequency. Typical values are for an rf power of 20 mW, a Ramsey interrogation time *T* = 100 s, and a static magnetic flux density *B*_static_ = 3×10^−7^ T. Here, *B* is the flux-density component at frequency *Ω*/2*π*

Physical effect	Scaling	Bias (×10^−15^)	Type B uncertainty (×10^−15^)
Quadratic Zeeman (static)	+ 〈*B*_static_^2^〉	20	1.4
Quadratic Zeeman (*Ω*)	+ 〈*B*^2^〉	5	3.2
Microwave chirp, leakage, and spectrum asymmetries	1/*T*_R_	3	0.8
